# Professor Dickson’s Introductory Lecture

**Published:** 1847-02

**Authors:** 


### PROFESSOR DICKSON'S INTRODUCTORY LECTURE.

We have been favored by the distinguished Professor of the *Institutes and Practice of Medicine*, in the Medical College of the State of South Carolina, with a copy of his Introductory Lecture, delivered at the opening of the present session. Its drift is the *inherent* claims of the medical profession on the respect and gratitude of society, and the causes, also inherent in the profession of this country, of its failing to secure what it ought to receive. As he advances, he cuts right and left, and woe be to him on whom the sabre falls.

He maintains, it is true, that in the *practice* of physic and surgery, the profession of the United States, have done as much as has been accomplished elsewhere; but sees many obvious and glaring defects, (growing chiefly out of our state of society,) that give a downward tendency to the profession, which will not, without great effort, be arrested. These defects lie chiefly in the want of preparatory education in our pupils, the lax and limited courses of study through which they are conducted in our schools, and their early and easy attainment of the doctorate. But on this point we must allow him to speak for himself:

"It is a grievous charge against the profession in these United States that its highest nominal rank is attainable at such a low rate of scientific acquirement, and with so little expenditure of time and labor. One cannot help being struck with the immense difference in this regard between us and our transatlantic brethren. In France, the candidate must attend four courses of lectures at least, upon all the various branches of medical science, aud undergo three examinations, some of the answers at which must be written extemporaneously, and in full detail. The "preliminary literary guarantees" demanded of pupils are a Bachelor's deg ee in Arts, and a Bachelor's degree in Sciences. They presuppose a classical and scientific education of ten years' duration. Yet the Medical Congress which lately met in Paris, a most august and imposing body, while they express their approbation of these preliminary guarantees as "sufficient but not too onerous," recommend as expedient that the duration of medical studies now occupying a period of *four* years be prolonged to *five;* that an examination be exacted at the end of *each year;* and that all students be obliged to serve for at least *a year* as dressers in a hospital.

"In England, the degree of Doctor of Medicine is not to be reached with less than eight years of study. At the University of London, the most liberal of the English schools, the student cannot even enter without an examination; he cannot be matriculated unless he "shows a competent knowledge of the classics, including the English languege, history and geography, mathematics and natural philosophy or chemistry." Before graduating as Doctor, he must have passed two examiations for the Bachelor's degree, and "a third, which is final;" exhibiting his attainments not only in "physiology, general pathology, general therapeutics, hygiene, surgery, practice of physic, midwifery and forensic medicine, or medical jurisprudence, but also in logic, and intellectual and moral philosophy." The number of persons ndmitted last year to the Doctorate of Medicine in England, was not more than 77; in our own country it amounted to fully 1,000! Within the last three years, the whole number of those licensed to practise as physicians, surgeons and apothecaries in England and Wales, is stated by the Medical Gazette at 2,721, being an annual average of 907. It is impossible to ascertain how many among us procure or assume the license to practice, which is indeed almost absolutely without restraint or limit.

It is, I fear, entirely too late even if it were not out of place here to discuss the question of the propriety or impropriety, the good or evil of this unrestricted system. The movement party are everywhere in the ascendant, the conservative principle almost extinguished; and we are going on to experiment freely upon the possibility of an existence untrammelled by any of the forms, which, in the Old World, are associated with recollections of inequality and oppression. In our vast republic, men shall hereafter preach, litigate and prescribe ad libitum; and the monopoly of skill, of preparation, and of diligence, no longer lie heavy on the bosom of the community. It is psssible that these tendencies shall be arrested—but the struggle is almost hopeless. On this downward road, as on the path to the den of Cacus, *nulla vestigia retrorsum.* In the meanwhile it behoves the profession, totally unprotected as it is likely to be, by any legislative countenance, to consider how it shall preserve itself from utter prostration and annihilation. We cannot require, as I have admitted, any high standard of preparatory education—the "preliminary literary guarantees" of the French system—but to compensate for this defect, we are bound to insist on a long-protracted and extensive course of technical studies. If human life and human health be worth consideration at all, and if professional ignorance can injure, and professional expertness can benefit mankind—questions which I cannot stoop even to entertain in this place, but shall assume the affirmative to be as clearly true as any proposition that can be submitted in language—then we should do all that we possibly can do to make the acquisition of knowledge certain, easy, full, nay, unavoidable. And how else can we effect this than by multiplying the opportunities presented to the student, and keeping him long in contact with the sources of information. Our systems must be accommodated to the wants and requirements of the average mind—the ordinary capacity. Talents and assiduity run rapidly forward and promptly overcome whatever difficulties lie in their way; but we must legislate also for dulness and indolence. This must be aided and stimulated. 'Repetition—repetition,' said the learned Wyttembach, 'is to the scholar what action, action, action is to the orator;' and by frequent repetition, the dullest will be instructed, and the slowest urged onward. Besides this, it never should be forgotten that whatever is caught up hurriedly is apt to be slightly impressed on the understanding and the memory; while acquisitions, more deliberately made, fix themselves with greater permanence, and stand out ever after with clear distinctness in the mind. I have already drawn a sketch of our American mode of study—text books read exclusively, or almost exclusively, cursorily, hastily; two half courses or one brief course and a half of lectures attended—listened to perhaps; a single dead body dissected under the eye of a Demonstrator, perhaps an arm only or a leg; a few surgical operations witnessed, such as may accidentally present themselves within the six months spent at the colleges; a few patients seen in the wards of a hospital at intervals of days, or it may be weeks. Our schools generally require three years of such study; but how seldom is even this rule complied with. The standard is so low, that any common industry may overleap it in half the time. We demand the legal age of maturity to be attained before the reverend Doctorate shall be reached—but our transatlantic youth, like the country which gives him birth, are of so quick and precocious growth, that they are universally ripened into grave manhood at or before twenty, if the family Bible and the parish register are to be trusted. There are many honorable exceptions I am aware—but I appeal to those who hear me, whether I color or exaggerate in the picture I have thus drawn.

As a predisposing remedy for these evils our author looks with interest to the approaching National Convention of Physicians in Philadelphia, but we fear will look in vain. Nevertheless, the Convention should be well attended, and all plans of reform and elevation freely, frankly, and forcibly discussed.

Want of space prevents our making further extracts, and we have so often presented our own views of the means of improving the profession of this country, that we shall not again obtrude them at this time. We hope the able lecture of the Southern professor will be extensively read in the region where he teaches with so much distinction, and that it may contribute to the amelioration which he has so much at heart.

